# Echinacoside protects against MPTP/MPP^+^-induced neurotoxicity via regulating autophagy pathway mediated by Sirt1

**DOI:** 10.1007/s11011-018-0330-3

**Published:** 2018-11-13

**Authors:** Chang Chen, Baomei Xia, Lili Tang, Wei Wu, Juanjuan Tang, Yan Liang, Hui Yang, Zhennian Zhang, Yan Lu, Gang Chen, Ye Yang, Yang Zhao

**Affiliations:** 1 Department of Neurology, The Third Affiliated Hospital of Nanjing University of Chinese Medicine, Nanjing, Jiangsu China; 2 Faculty of Rehabilitation Science, Nanjing Normal University of Special Education, Nanjing, Jiangsu China; 3 Physiology Research Section, School of Medicine and Life Sciences, Nanjing University of Chinese Medicine, Nanjing, Jiangsu China; 4 Center for Translational Systems Biology and Neuroscience, School of Basic Biomedical Science, Nanjing University of Chinese Medicine, Nanjing, Jiangsu China; 5 Center for Modernization of Chinese medicine and database, The Third Affiliated Hospital of Nanjing University of Chinese Medicine, Nanjing, Jiangsu China

**Keywords:** Parkinson’s disease, Echinacoside, Autophagy, Sirt1

## Abstract

Parkinson’s disease (PD) is a common chronic neurodegenerative disease and greatly affects the quality of PD patients’ life. Current symptomatic treatment of PD is limited. There are no effective treatment and drugs that could radically cure PD. Increasing experimental evidence has proven a causal relationship between alpha-synuclein (α-synuclein, α-syn) and the neuropathology of Parkinson’s diseases, although the exact pathophysiological role of α-synuclein is not fully clarified. Previous studies showed that monomers and polymers of α-synuclein were secreted from damaged nerve cells via exocytosis and occupied healthy nerve cells via endocytosis, which afford evidence for the prion-like role of α-synuclein. Autophagy is the known mechanism for eukaryotic cells to degrade protein polymers and damaged organelles that proteasome does not cope with. Therefore, promoting the clearance of α-synuclein by enhancing autophagy in neuronal cells could be a promising treatment in the early stage of PD. SIRT1 is a potent regulator of autophagy, because it deacetylates a mass of important transcription factors such as Forkhead Box subgroup O (FoxO) transcription factors family. SIRT1’s action relates to FoxO, because FoxO transcription factors are involved in various molecular pathways underlying neuronal protection and autophagy. Moreover, Sirt1 deacetylates proautophagic proteins such as Atg5, Atg7, and Atg8. Echinacoside (ECH) is the main active ingredient of a widely used Chinese herb cistanche, which has been proven to elicit neuroprotective effects in models of neurodegenerative diseases. In this study, we found that ECH could improve PD-like symptoms in MPTP-lesioned mouse model. We further showed that the underlying mechanism of the action of ECH was associated with enhancing autophagy in neurons via bind to Sirt1 directly and affect FoxO expression. Our study demonstrated ECH as a potential therapeutic agent against PD.

## Introduction

Neurodegenerative disorders exert a heavy toll on patients’ quality of life and pose an economic burden for society. Parkinson’s disease (PD) ranks second most common neurodegenerative disease after Alzheimer disease. Until now, no medications have been proved to be efficacious in slowing progression of PD. Therefore, any potentially disease-modifying treatments have been met with great enthusiasm (Stoessl 2017).

Currently, increasing dopamine concentrations or directly stimulating dopamine receptors as the drug therapeutics, are the main treatment measures of Parkinson’s disease. However, there are many disabling characteristic including motor symptoms that do not react to dopaminergic therapies or complications of long-term dopaminergic drug use, as well as a series of non-motor symptoms. Up to now, treatment measures that reduce neurodegeneration or prevent the disease process have remained elusive (Kalia and Lang 2015).

Parkinson’s disease is characterized by early prominent death of dopaminergic neurons in the substantia nigra pars compacta (SNpc). The presence of SNpc degeneration and Lewy pathology in post-mortem pathological examination are considered as the gold criteria for the diagnosis of PD. Lewy pathology is constitutive of abnormal aggregates of α-synuclein protein, called Lewy bodies and Lewy neurites (Kalia and Lang 2015). PD is thought to be associated with environmental factors such as pesticides and herbicides, while MPTP (1-methyl-4-phenyl-1,2,3,6-tetrahydropyridine) has a similar structure to them (Ahmed et al. 2017). It is proved that MPTP could induce obvious neurotoxicity of both pathological and biochemical damage dopaminergic neurons in the nigrostriatal pathway (Abushouk et al. 2017). Therefore it becomes the most commonly used neurotoxin for the preparation of PD animal model.

Sirtuins are nicotinamide adenine dinucleotide (NAD^+^)-dependent deacylases which is traditionally associated with calorie restriction and mammalian aging. As well, these proteins play important roles in maintaining health of neurons during aging. In the course of neuronal development, the SIR2 ortholog SIRT1 plays an important role in structure, promoting axonal elongation, neurite outgrowth, and dendritic branching (Herskovits and Guarente 2014).

In addition to its important role of normal brain aging process, SIRT1 has also been shown to improve a series of neurodegenerative disorders in animal models including Alzheimer’s, Parkinson’s, and Huntington’s disease. A number of studies have demonstrated the neuroprotective effect of SIRT1 in vivo and in vitro PD models. Several mechanisms have been identified. First, SIRT1 can promote the transcription of heat shock protein 70 and other molecular chaperones by deacetylating heat shock factor 1. Another possible mechanism is that SIRT1 deacetylate PGC1a as overexpressed PGC1a or resveratrol to protect dopaminergic neurons from MPTP-induced cell degeneration. SIRT1 may also regulate autophagy and mitotic signaling pathways, thereby reducing the toxicity of α-synuclein in PD (Herskovits and Guarente 2014).

Echinacoside (ECH) is derived from the fleshy stem of cistanche, which has known beneficial effects such as bone protection (Yang et al. 2013), liver protection (Wu et al. 2007), antioxidant (Wu et al. 2007), anti-inflammatory (Jia et al. 2014), anti-aging (Xie et al. 2009), and other pharmacological effects. Recent studies have found that ECH can penetrate blood-brain barrier, therefore effectively protect the central nervous system against neurodegeneration (Zhu et al. 2013). ECH elicited neuroprotective effects in subacute MPTP induced-PD mouse model (Zhao et al. 2010) through increasing the expression of neurotrophic factors and inhibited apoptosis. ECH can protect neuronal cells against rotenone injury through suppressing activation of Trk-ERK signal pathway and decreasing the release of cytochrome C and caspase-3 (Zhu et al. 2013). In the study of Y Zhang et al., ECH relieved endoplasmic reticulum stress induced by 6-OHDA and rescued damaged neurons through mediated Grp94/Bip-ATF4-CHOP signal pathway (Zhang et al. 2017b). Moreover, ECH protected 6-OHDA-induced striatal dopaminergic neurons injury in rats (Chen et al. 2007). The exact mechanism of ECH-induced neuroprotection effect in PD is not fully understood.

In the preliminary stage, our research team performed molecular docking by adopting structure-based drug design and predicted that ECH molecule could directly combine with Sirt1 protein to exert neuroprotective effect. This study focuses on MPTP-induced PD mouse model and MPP^+^ induced PC12 cell model to explore how ECH regulates Sirt1 and how it performs neuroprotective effect in PD in vitro and in vivo models.

## Materials and methods

### Materials

Echinacoside (ECH, CAS Number 82854–37-3), 1-Methyl-4-phenyl-1,2,3,6-tetrahydropyridine hydrochloride (MPTP, M0896), 1-Methyl-4-phenylpyridinium iodide (MPP^+^, D048), 3-(4,5-Dimethyl-2-thiazolyl)-2,5-diphenyl-2H-tetrazolium bromide (MTT, M2128) and Chloroquine diphosphate salt (CQ, C6628) were obtained from Sigma-Aldrich. EX527 (HY-15452) was purchased from MedChemExpress. DMEM and foetal bovine serum were purchased from Gibco.

### Animals and treatments

Ten-week-old Male C57BL/6 J mice weighing 25-28 g were used in this Experiments. The animals were kept under a conditions of 12/12 h light/dark cycle, 21 ± 2 °C and were given free access to food and water. All animal procedures conformed to the Guide for the Care and Use of Laboratory Animals and were approved by the Institutional Animal Care and Use Committee at Nanjing University of Chinese medicine. The experimenters were blinded to the assignments of the mice.

**Fig. 1:**
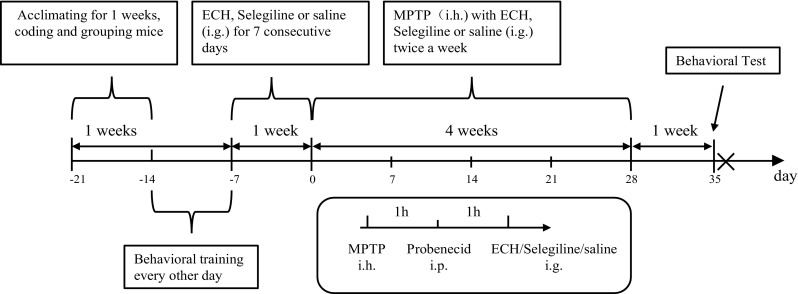
Experimental time axis. A timeline elaborating the experimental process including the time of acclimatization, coding, grouping, behavioral training, drug treatments and time points for behavioral tests

After being housed in animal facilities for 1 week to acclimate, mice were randomly divided into the four groups (10 mice per group): group A (vehicle control group, an equal volume of normal saline(NS)); group B (MPTP model group, treated with an equal volume of saline); group C (treated with 30 mg/kg Echinacoside (Chengdu Research Institute of Biology of the Chinese Academy of Sciences, HPLC≥98%,82,854–37-3); groups D (positive control group, treated with 30 mg/kg Selegiline (Finland Orion Corporation). Four groups of animals were pre-trained for behavioral test every other day in the next week. All groups were administered the respective pre-treatment compounds orally (p.o.) every 24 h for 7 consecutive days, with the last day of administration designated as day 0. Then the mouse PD models for groups B, C, D were made by injecting MPTP (sigma, M0896) at a dose of 30 mg/kg twice a week. Probenecid (250 mg/kg) was injected intraperitoneally 1 h following subcutaneous injection of MPTP to delayed its metabolism, and 1 h later respective treatment compounds were oral administration. Groups A received the same administration schedule except an equal volume of saline was injected instead of MPTP. All the animals were sacrificed at the 36th day post the behavioral test (Fig. 1).

### Pole test

To assess bradykinesia and motor coordination, the pole test was implemented according to previously published methods (Drucker-Colín and García-Hernández 1991). Selecting a steel pipe (55 cm long, 1 cm in diameter) tightly wrapped in white antiskid tape. Placing a spherical protrusions (2 cm in diameter) on the top of the pipe as attachment points of mice. The pipe should be fixed on the plastic foam base and placed in a cage with thick dressing. Putting mice on the upside of the spherical protrusions and recording the total time (T-total) of climbing to the bottom.

### Cell culture

PC12 cells were cultured in 10% foetal bovine serum DMEM, 100 U/ml of penicillin and 100 μg/ml of streptomycin at 37 °C under a containing 5% CO_2_ humidified atmosphere. When the cells enter logarithmic growth phase, they were seeded into 96-well plates at a density of 1 × 10^4^/well for 24 h. In order to determine the non-toxic dosages of ECH, PC12 cells were incubated with ECH (12.5-300 μM) for another 24 h. To study the effect of ECH on cell viability in MPP^+^-treated PC12 cells, the cells were pre-cultivated in serum-free DMEM with different concentrations of ECH (12.5-300 μM) for 1 h. Subsequently, MPP^+^ was added to the wells at a final concentration of 1 mM and incubated for another 24 h. CQ or EX527 was added 2 h before ECH.

### Cell viability assay

Cell viability was measured using an MTT assay (Sladowski et al. 1993; Lv et al. 2017). After the initial incubation, a concentration of 0.5 mg/ml MTT was added to each well, then incubating for further 4 h at 37 °C. After removing the supernatant, the formazan product at bottom was solubilized sufficiently with 100 μl dimethyl sulfoxide (DMSO). The solutions was measured at 570 nm optical density using Epoch microplate assay reader (Bio-Tek).

### Hoechst staining

PC12 cells were seeded into 24-well plates for 24 h before changing the culture medium and random grouping. ECH(100 mM)was pre-treated 1 h following the CQ which was pre-dosage of 0.05 μM, and 30 min later incubating with MPP+ (1 mM) for 24 h, change for serum-free DMEM medium and add Hoechst 33342 solution with a final concentration of 10 μg/mL for 5 min at room temperature. Subsequently, abandon the culture medium and add formaldehyde solution with a concentration of 4% for another 10 min. Then wash the cells 2 times with 1 mL PBS solution, each time for 5 min. Finally, observe the size and shape of cell nucleus under ultraviolet filter of Olympus fluorescence microscope (excitation wavelength is 360 nm and scattering wavelength is 420 nm) to determine and reflect apoptosis.

### Immunohistochemical detection (Lv et al. 2017)

After the left ventricle perfusion, the mice were quickly taken to the brains and placed in a 4% paraformaldehyde. After the gradient dehydration of sucrose solution, brains was buried with OCT adhesive, and the frozen slicer was used as a continuous slice of brain tissue (30 μm thick). Slices of interest were selected from the SN-VTA according to the atlas of Paxinos and Franklin (2004). Frozen sections from the substantia nigra were incubated with anti-TH antibody (Sigma, SAB4200697, 1: 1000) in blocking solution (0.3% *v*/v Triton X-100 and 10% v/v BSA in PBS) for 12 h. Next, the slices were washed three times in 0.01 M PBS for 10 min each time. A second incubation with goat anti-mouse IgG H&L (HRP) (Abcam, ab6789, 1:2000) was performed for 2 h at 25 °C. After several washings with PBS, the antibody complex was detected using a modification of the ABC system (Kit Vectastain ABC, Vector Laboratory Inc.). The midbrain dopaminergic cell groups were plotted from TH-immunostained slices. TH-immunoreactive (TH-ir) neurons were stereologically quantified using Image-Pro Express 6 (Media Cybernetics). The mean number of TH positive neurons in each hemisphere, obtained through the quantification of four alternating slices, was considered to be representative of the SNpc neuronal cells in each animal. The selected areas were digitized with a microscope (DX45; Olympus). The images were taken using an Olympus camera (DP72; Olympus) at an original magnification of 100 × .

### Western blotting (Yang et al. 2018)

Substantia nigra and PC12 cells were prepared in RIPA buffer containing protease and phosphatase inhibitors. Protein concentration was measured by BCA assay (Pierce). 20 μg of total protein from each sample was separated by SDS-PAGE (8%, 10% or 15%) and transferred to a PVDF membrane. After incubating in 0.01 M TBS, 0.1% Tween-20 and 5% non-fat dry milk powder for 1 h to block any remaining protein binding sites, membranes were incubated overnight at 4 °C using the following primary antibodies: the mouse antibody against TH (Sigma, SAB4200697, 1:2000), alpha-synuclein (Abcam, ab1903, 1:1000), β-tubulin (Proteintech,10,094–1-AP, 1:2000); the rabbit antibody against LC3, P62, Beclin-1, p-PI3K, PI3K, Sirt1 (Cell Signaling Technology, #4108, #5114, #3738, #4228, #4249, #9475, 1:1000) and FoxO1 (Abcam, ab52857, 1:1000). After three 5-min washes in TBST, membranes were incubated with the goat anti-mouse IgG H&L (HRP)/goat anti-rabbit IgG H&L (HRP) (Abcam, ab6789, ab6721, 1:2000) for 1 h at room temperature. All antibody incubations and washing steps were carried out in TBST. The immunoreactive blots were visualized using the supersignal chemiluminescence detection system (Thermo Fisher Scientific Inc.) and quantified with densitometry using ImageJ software.

### Statistical analysis

Data were analyzed using SPSS Version 20.0 (IBM). All experiments were performed at least three times. Comparisons between two groups were performed using a two-sample Student’s t test, and comparisons between multiple groups were performed using a one-way ANOVA followed by Tukey’s post hoc test. All data are presented as mean ± SEM, and statistical significance was accepted at the 5% level unless otherwise indicated.

## Result

### Echinacoside reversed the behavioral impairments and attenuated the loss of nigral TH-positive neurons in chronic MPTP-lesioned mice

**Fig. 2:**
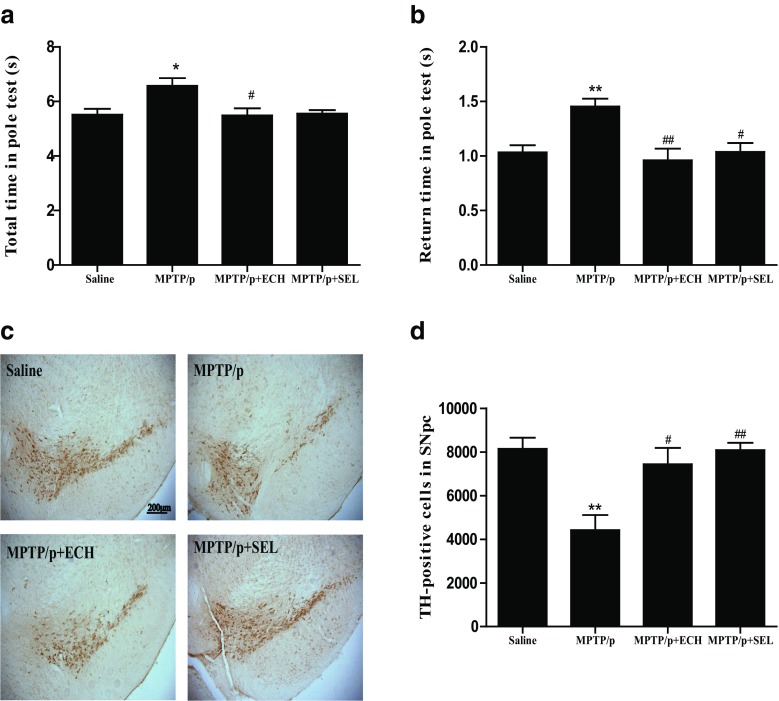
Behavioral effect of in pole test by ECH in PD model mice. ECH improved MPTP-induced bradykinesia and coordination in the pole test. **a** Total time (sec); **b** return time (sec). *n* = 6–7/group. *p < 0.05, **p < 0.01, compared with the Saline group; #*p* < 0.05, ##*p* < 0.01, compared with the MPTP group. Photomicrographs and quantification of TH positive cells in SN-VTA of MPTP-induced mice and the effects of ECH treatment. **c** Representative microphotographs of dopaminergic neurons stained for TH. ECH rescued the loss of nigral TH positive neurons induced by MPTP. Scale bars: 200 μm. **d** The quantification of TH+ cell in each group. *n* = 4/group. Values represent means ± SEM. **p < 0.01, compared with the Saline group; #*p* < 0.05, ##*p* < 0.01, compared with the MPTP/p group

We used the pole test to evaluate bradykinesia and coordination in mice. At the seventh day after the last induction of MPTP, we detected that the return time and the total time were both significantly prolonged, and the effect was significantly reversed with ECH and selegiline treatment (Fig. 2a, b). Our results suggested that ECH reversed the behavioral impairments induced by MPTP. Then we used immunohistochemistry staining for TH-positive neurons to observe the loss of dopaminergic neurons in four groups of mice. As shown in Fig. 2c and d, MPTP inducement reduced the number of nigral TH positive neurons remarkably. Compared with the MPTP group, the administration of ECH and selegiline significantly increased the number of nigral TH positive neurons.

### Echinacoside inducted autophagy and enhanced the degradation of α-synuclein of MPTP-treated mice

**Fig. 3:**
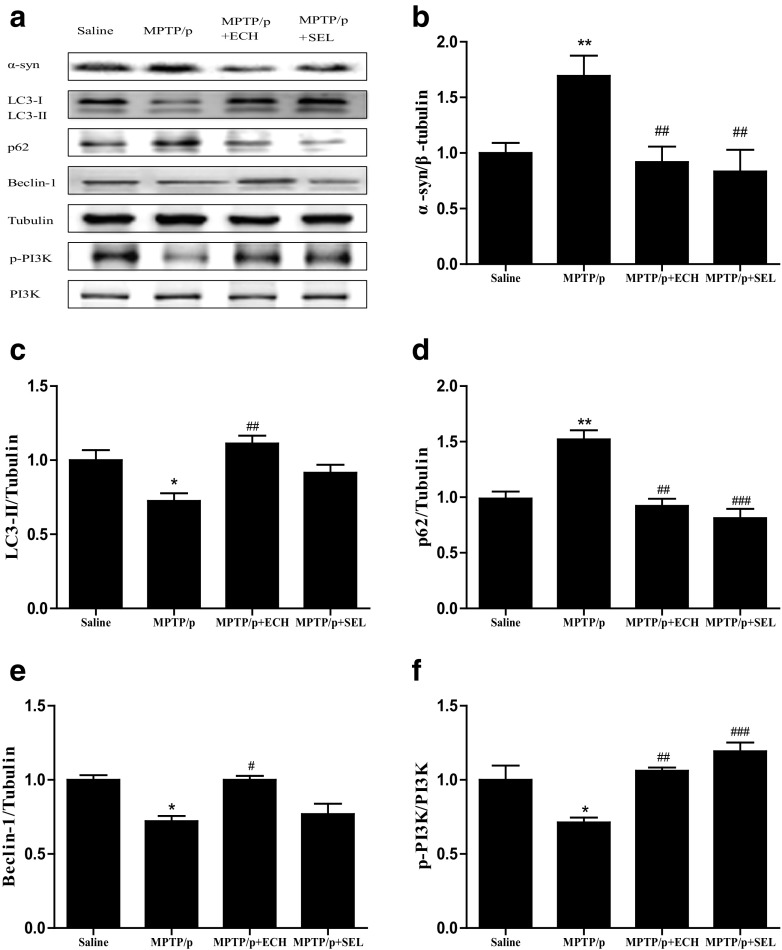
The effect of ECH on autophagy protein and α-synuclein in PD mice. The substantia nigra protein expressions of LC3, p62, Beclin1, p-PI3K, PI3K and α-synuclein. Tubulin protein act as the internal control. Values are presented as means ± SEM (*n* = 3–4). *p < 0.05, **p < 0.01, compared with the Saline group; #*p* < 0.05, ##*p* < 0.01, ###*p* < 0.001, compared with the MPTP group

Western blotting analysis indicated that MPTP-induced a marked α-synuclein protein increase in striatum tissue compared to saline control (7 days after last injection). However, the MPTP-lesioned animals treated with ECH and selegiline inhibited the accumulation. Meanwhile, after MPTP treatment, the expression of autophagy-related proteins such as LC3-II, Beclin-1 and p-PI3K reduced while the expression of P62 which is negatively related to autophagy significantly increased (*p* < 0.05), showing that the level of autophagy in neurons reduced after modeling. ECH could reverse such change by increasing the expression of LC3-II, Beclin-1 and p-PI3K and reducing the expression of P62 (Fig. 3). Taken together, our results suggested that the mechanism of ECH in alleviating loss of DA neurons and increase of α-synuclein in SNpc induced by MPTP is related to up-regulation of autophagy.

### Echinacoside protected the viability of MPP^+^ induced PC12 cells by increasing autophagy

**Fig. 4:**
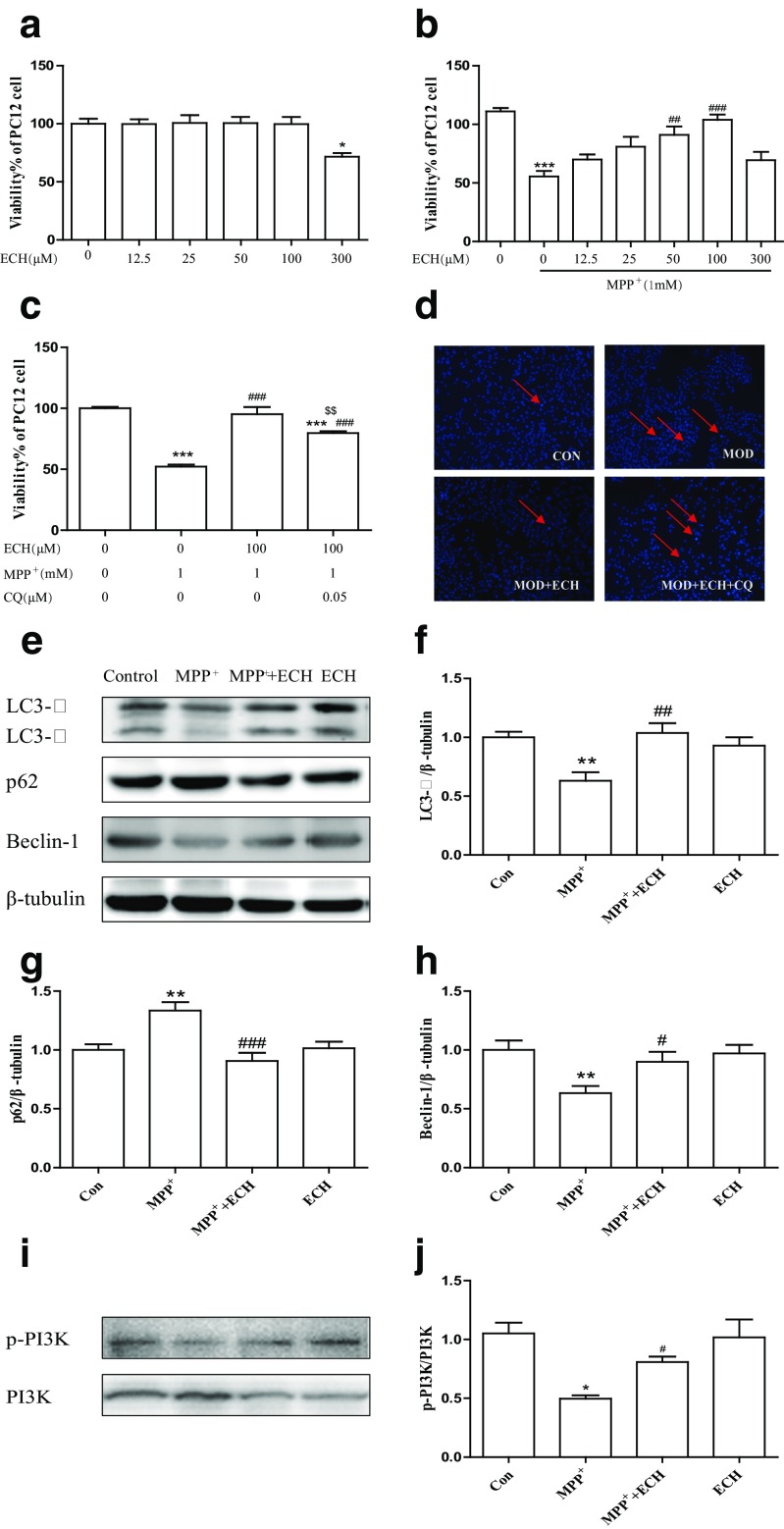
ECH protected the viability of MPP^+^ induced PC12 cells by increasing autophagy Protective effect of ECH on viability of PC12 cells injured by MPP+. Cell viability was determined by MTT assay. Data are expressed as percentage of the viability of cells, control taken as 100% viability. **a** Effect of different concentration of ECH on cell viability in PC12 cells (determine the non-toxic dosages of ECH). PC12 cells were incubated with ECH (12.5–300 μM) for 24 h. **b** Effect of ECH on cell viability changes in MPP^+^-induced PC12 cells. PC12 cells were pre-incubated with different concentration of ECH (0, 25, 50, 100 μM) for 1 h. Then, MPP+ was added to the wells at a final concentration of 1 mM and incubated for another 24 h at 37 °C. **c** Protective effect of ECH on cell viability was reduced by CQ. Values are presented as means ± standard error (*n* = 6). *p < 0.05, **p < 0.01, ***p < 0.001, compared with the con group; #*p* < 0.05, ##*p* < 0.01, ###*p* < 0.001, compared with the MPP^+^ group. **d** Apoptotic cells were examined in terms of changes in cell morphology by Hoechst 33342 staining (red arrows were represented as apoptotic cells). **e**‑**j** Western blotting analysis and quantification of relative LC3, p62, Beclin1, p-PI3K and PI3K protein abundance in the PC12. Tubulin protein served as the internal control. Values are presented as means ± standard error (*n* = 3). *p < 0.05, **p < 0.01, compared with the con group; #*p* < 0.05, ##*p* < 0.01, ###*p* < 0.001, compared with the MPP+ group

In this study, we showed ECH has an effect of protection on neurons. We further tested the cell viability of PC12 cells treated with different concentrations of ECH to determine non-toxic dosages, as shown in Fig. 4a. There was no significant difference been observed in the viability of PC12 cells with concentrations of 0-100 μM of ECH treatment. However, cell viability was significantly lower at a concentration of 300 μM than the other groups of concentrations. To determine whether ECH had neuro-protective effects against the administration of MPP^+^, PC12 cells were pre-incubated with the concentrations of 0-300 μM ECH for 1 h followed by exposing to 1 mM MPP^+^ for 24 h. The MTT assay showed that treating PC12 cells with MPP^+^ alone resulted in a 40% reduction in the number of surviving cells. Co-treatment with 50 or 100 μM ECH showed a reduction in MPP^+^-induced cytotoxicity. PC12 cell viability increased in an ECH-dose-dependent manner compared with the cells treated with MPP^+^ only (Fig. 4b). This indicates that ECH reduced MPP^+^-induced cytotoxicity. Consequently, we chose to use concentrations of 100 μM of ECH in the following experiment. To confirm the enhancement of autophagy by ECH, we treated MPP^+^-induced PC12 cells with ECH and ECH plus CQ (chloroquine, a lysosome inhibitor). Then the protective effect of ECH on MPP^+^ induced PC12 cell damage was reduced (Fig. 4c, d). Western blotting detection indicated that the expressions of autophagy proteins such as LC3-II and Beclin-1 and p-PI3K were significantly reduced (*p* < 0.01, *p* < 0.01, *p* < 0.05) while the expression of P62 which is negatively related to autophagy was significantly increased (*p* < 0.05) in the PC12 cell after being incubated with 1 mM MPP^+^ for 24 h. 100 μM ECH treatment increased the expression of LC3-II, Beclin-1 and p-PI3K (*p* < 0.05, *p* < 0.01, *p* < 0.05) while reduced the expression of p62 (*p* < 0.05). (Figure 4e‑j) The result above indicated that ECH reversion of MPP^+^ induced PC12 cell damage was related to the up-regulation of autophagy.

### Echinacoside could bind to Sirt1 and up-regulated the expression of Sirt1 and FoxO1 in MPP^+^ induced PC12 cells

**Fig. 5:**
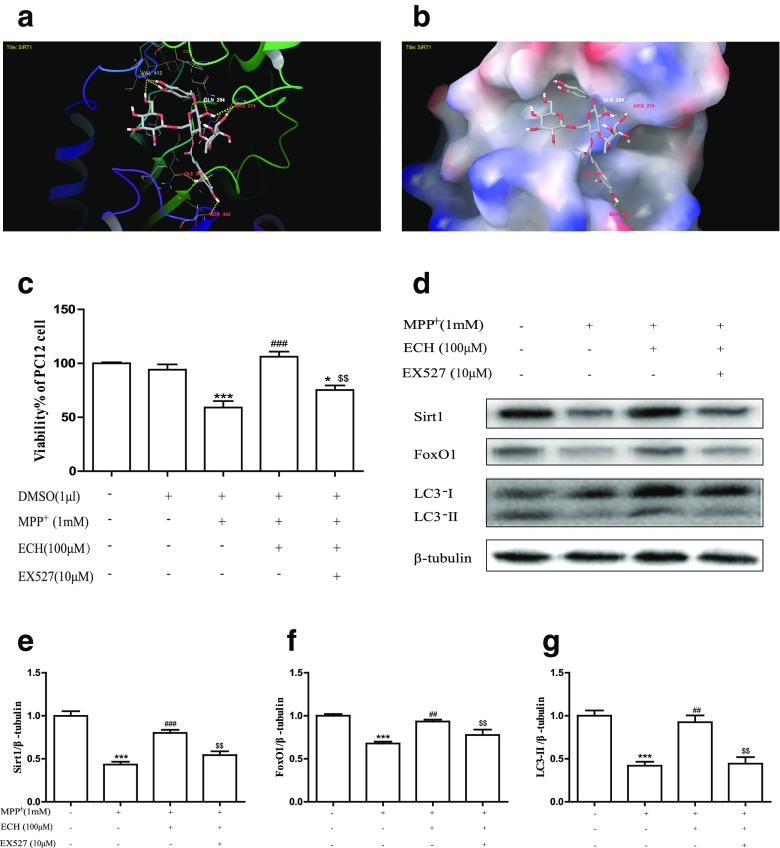
ECH bind to Sirt1 and up-regulated expression of Sirt1 and FoxO1 in MPP^+^ induced PC12 cells. **a** Docking pattern of the protein Sirt1 and the ligand ECH. **b** Sirt1 protein surface ligand docking ECH schematic. The protein surface of Sirt1 was calculated from the electrostatic energy of the amino acids. **c** Protective effect of ECH on cell viability was reduced by EX527. **d**‑**g** Alternations of expression on Sirt1, FoxO1 and LC3 protein in the PC12. β-Tubulin protein served as the internal control. Values are presented as means ± standard error (*n* = 3). *p < 0.05, ***p < 0.001, compared with the con group; ##*p* < 0.01, ###*p* < 0.001, compared with the MPP+ group; $$*p* < 0.01, compared with the MPP^+^+ECH group

Structure-based drug design was adopted to evaluate whether Sirt1 can bind to ECH and forecast their docking pattern and docking energy. Preparations including adding hydrogens and charges were performed to Sirt1 and ECH respectively before docking the ligand ECH to the receptor protein Sirt1. Result of the molecular docking revealed that the docking energy of ECH to Sirt1 was −12.5 kcal/mol, and the micromolecule and protein have 9 hydrogen bonds, forming a stable docking pattern (Fig. 5a). ECH molecules falls inside the active pocket of protein Sirt1 (Fig. 5b). The calculation forecasted that ECH molecules could directly bind to protein Sirt1. The result above indicated that ECH and Sirt1 have affinity, and ECH was likely to regulate the pharmacological effect of Sirt1. Furthermore, we use EX527 (specific Sirt1 inhibitor) to validate this conjecture. We found that the protective effect of ECH on MPP^+^ induced PC12 cell damage was reduced by EX527 (Fig. 5c). Followed by the western blotting, Sirt1 and FoxO1 (the downstream protein of Sirt1) protein expression were significantly down-regulated after MPP^+^ exposure on PC12, and ECH reversed this phenomenon. However, when EX527 was applied, the effect of ECH was reduced. Meanwhile, the effect that ECH raised LC3 was also reduced. (Fig. 5d‑g). The experiment showed that the enhancing autophagy effect of ECH may be achieved by regulating Sirt1.

## Discussion

Parkinson’s disease is defined as a common and complex neurological disease. It is now considered to be a slow progressive neurodegenerative disorder that touches on numbers of neuroanatomical areas, and shows a wide range of symptoms. Research and development of disease-modifying drugs which could slow or prevent the neurodegenerative process is a main objective of PD research. The pathological change of PD is characterized by the presence of SNpc degeneration and the generation of Lewy Body (LB) pathology. LB is a characteristic marker of Parkinson’s disease, and its main components are aggregated and misfolded fibrous alpha-synuclein (α-synuclein, α-syn). Alpha-synuclein, mainly expressed at presynaptic terminals in the central nervous system, is a small soluble protein. Although the association between LB and pathogenesis of PD is poorly understood (Kalia and Lang 2015), strong evidence has displayed that aggregation of α-synuclein contributes to dopaminergic cell loss in the disease (Lo Bianco et al. 2002; Recchia et al. 2004). Therefore, promoting the clearance of α-synuclein is viewed as a potential therapeutic method for treating PD (Lashuel et al. 2013).

MPTP is not toxic itself, but it can cross the blood brain barrier and then be transformed by monoamine peroxidase enzyme to MPP^+^, which combines with dopamine transporters (DAT), leading to inhibition of dopamine uptake and depletion of its cerebral levels. Therefore, experiments on MPTP induced animal models have a great advantage in terms of efficacy evaluation as compared to other PD models. And it leads to better understanding of PD pathology including microglial activation and oxidative stress. Besides, inhibition of alpha-synuclein degradation is also a major neurotoxic mechanism of MPTP (Abushouk et al. 2017).

**Fig. 6:**
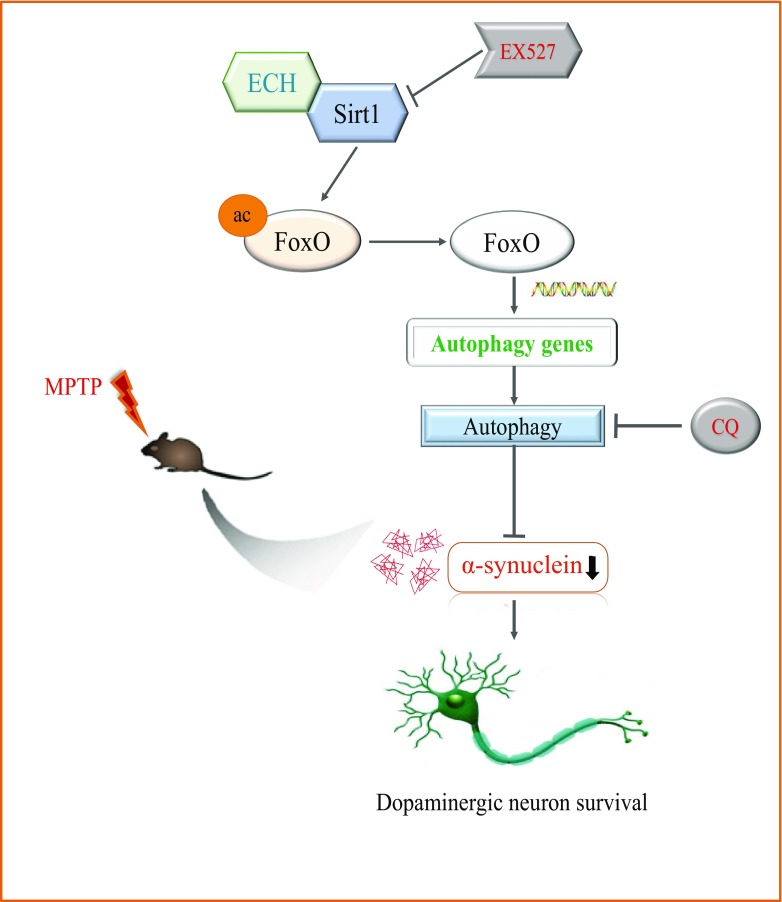
A graphical illustration of the hypothesis. ECH combined with SIRT1 (while EX527 inhibited SIRT1), which activated FoxO1, leading to autophagy gene transcription and translation. The phenomenon promoted the autophagic degradation on α-synuclein (while CQ inhibited the autophagic degradation), which reversed the MPTP-induced loss of dopaminergic neurons

In this study, we found that ECH could reverse MPTP induced dopaminergic neurodegeneration and behavioral impairments. We have also demonstrated that the protective effect of ECH was achieved by activating autophagy pathway, subsequently, Sirt1 mediates the autophagic degradation of α-synuclein by activating FoxO1 (Fig. 6).

Echinacoside is a kind of benzoside isolated from the stems of the traditional Chinese medicine, Cistanche salsa, which is routinely used in traditional Chinese medicine formulas for the treatment of symptoms relevant to PD. It has many beneficial effects, such as nerve protection, liver protection, anti-inflammatory, anti-fatigue, anti-tumor, anti-oxidation and so on (Lei et al. 2001). Recently, ECH has been shown to have many neuroprotective effects and is considered to be one of the new compounds for the treatment of PD. Several studies have shown that it could significant improves motor behavior and inhibit the loss of nigral DA neurons in MPTP-lesioned mice (Zhao et al. 2010). Other studies have shown that ECH protected neurons by regulating the activation of the ROS/ATF3/CHOP pathway (Zhao et al. 2016) or by regulating the activation of p38MAPK and NF-κB p52 signals (Zhang et al. 2017a). In this study, we revealed that ECH has a protective effect against MPTP/p-induced neuron damage in nigrostriatal dopaminergic neurons through activating the autophagy pathway mediated by sirt1 in vivo and in vitro.

First, we used MPTP induced B6 mice to produce PD model and intervened with ECH. We found that the neurobehavioral changes of PD in mice resulted from MPTP was reversed by ECH while mice treated with ECH alone demonstrated no obvious difference from mice in the control group. We did not detect more PD-related behavioral test, because Zhao Qing, Geng Xingchao, etc., have detected the test including gait dysfunctions, spontaneous motor activity test, rotarod tests, and have proved that ECH did improve MPTP-induced PD sample behavior in mice (Zhao et al. 2010; Geng et al. 2007). Second, we detected α-syn, a typical pathological protein of PD, finding the increase of α-syn which could be partially reversed by ECH. This suggests that ECH may have disease-modifying effect on PD. We also verified the neuroprotective effect of ECH to PD model by producing PD cell model with 1 mM MPP^+^ induced PC12 cell. It showed cell deaths and it was reversed after pre-intervention of ECH with concentrations of 25, 50 and 100 μM. Subsequently, we verified the safety of three dosages by showing that there was no obvious cell proliferation and death after adding ECH with different concentrations into PC12, proving that ECH with these three concentrations has no cytotoxity to normal PC12 cells. On this basis, we chose ECH with a maximum safe concentration of 100 μM for subsequent experiments.

Then we adopted structure-based drug design to evaluate whether Sirt1 could bind to ECH and forecast their docking pattern and docking energy. We found that ECH could directly bind to Sirt1. It has been reported that Sirt1 has the neuroprotective effect in neurodegenerative diseases, which could depend on its function in lengthening cell lifespan and bolstering cell viability. Sirt1 is a nuclear protein expressed in a broad range of tissues including the brain (Ramadori et al. 2008). Sirt1 binds and deacetylate FoxO family transcription factors in brain (Donmez 2012). Because FoxO transcription factors are involved in many molecular pathways, such as neuronal protection, stress resistance and glucose production, part of the reason Sirt 1 participates in these mechanisms is FoxO (Salminen et al. 2008). It is known that overexpression of Sirt1 in PD animal models and cell models inhibited the formation of α-synuclein aggregates by activating molecular chaperones (Donmez and Outeiro 2013).

In our study, the expression of Sirt1 was significantly up-regulated in PD cells after ECH intervention, which predicted that the neuroprotective effect depends on the regulation of Sirt1. Recent studies have shown that Sirt1 was an effective autophagy degradation regulator because it interacted with deacetylation of autophagic proteins such as Atg5, Atg7 and Atg8 and so on (Lee et al. 2008).

The ubiquitin-proteasome system (UPS) and autophagy-lysosomal pathway (ALP) are the two most important ways to degrade aggregated or misfolded proteins such as α-synuclein. Recently, PD patients and PD animal models have been observed the defect in the autophagy pathway in the brains (Lynch-Day et al. 2012). Meanwhile, researchers hold a view that the clearance of aggregated α-synuclein depends on autophagy more than proteasome (Yelamanchili et al. 2011). These evidences manifest that autophagy plays an important role in PD and suggest that autophagy inducers might be a potential treatment for PD (Chen et al. 2014).

Further study found that the levels of autophagy in MPP^+^ induced PC12 cells and MPTP induced B6 mice midbrain were significantly declined but restored after ECH intervention. The effect of rescue was reduced by CQ and EX527. Therefore, we believe that ECH enhances autophagy pathway activation by binding to Sirt1 and performs neuroprotective function.

Echinacoside has pleiotropic actions, benefits and the identification of multiple related targets of action, just as ECH acts through multiple channels. This study shows that ECH improves the motor function deficits and pathological changes of PD mice by activating Sirt1, which led to FoxO1 up-graduation and mediates autophagic degradation of α-synuclein. Our research also proposed a mechanism to support ECH as a PD preventive or therapeutic agent.
